# Predicting Coronary Stenosis Progression Using Plaque Fatigue From IVUS-Based Thin-Slice Models: A Machine Learning Random Forest Approach

**DOI:** 10.3389/fphys.2022.912447

**Published:** 2022-05-10

**Authors:** Xiaoya Guo, Akiko Maehara, Mingming Yang, Liang Wang, Jie Zheng, Habib Samady, Gary S. Mintz, Don P. Giddens, Dalin Tang

**Affiliations:** ^1^ School of Science, Nanjing University of Posts and Telecommunications, Nanjing, China; ^2^ The Cardiovascular Research Foundation, Columbia University, New York, NY, United States; ^3^ Department of Cardiology, Zhongda Hospital, Southeast University, Nanjing, China; ^4^ School of Biological Science and Medical Engineering, Southeast University, Nanjing, China; ^5^ Mallinckrodt Institute of Radiology, Washington University, St. Louis, MO, United States; ^6^ Department of Medicine, Emory University School of Medicine, Atlanta, GA, United States; ^7^ The Wallace H. Coulter Department of Biomedical Engineering, Georgia Institute of Technology, Atlanta, GA, United States; ^8^ Mathematical Sciences Department, Worcester Polytechnic Institute, Worcester, MA, United States

**Keywords:** coronary atherosclerosis, stenosis prediction, IVUS, fatigue, random forest, patient-specific models

## Abstract

**Introduction:** Coronary stenosis due to atherosclerosis restricts blood flow. Stenosis progression would lead to increased clinical risk such as heart attack. Although many risk factors were found to contribute to atherosclerosis progression, factors associated with fatigue is underemphasized. Our goal is to investigate the relationship between fatigue and stenosis progression based on *in vivo* intravascular ultrasound (IVUS) images and finite element models.

**Methods:** Baseline and follow-up *in vivo* IVUS and angiography data were acquired from seven patients using Institutional Review Board approved protocols with informed consent obtained. Three hundred and five paired slices at baseline and follow-up were matched and used for plaque modeling and analysis. IVUS-based thin-slice models were constructed to obtain the coronary biomechanics and stress/strain amplitudes (stress/strain variations in one cardiac cycle) were used as the measurement of fatigue. The change of lumen area (DLA) from baseline to follow-up were calculated to measure stenosis progression. Nineteen morphological and biomechanical factors were extracted from 305 slices at baseline. Correlation analyses of these factors with DLA were performed. Random forest (RF) method was used to fit morphological and biomechanical factors at baseline to predict stenosis progression during follow-up.

**Results:** Significant correlations were found between stenosis progression and maximum stress amplitude, average stress amplitude and average strain amplitude (*p* < 0.05). After factors selection implemented by random forest (RF) method, eight morphological and biomechanical factors were selected for classification prediction of stenosis progression. Using eight factors including fatigue, the overall classification accuracy, sensitivity and specificity of stenosis progression prediction with RF method were 83.61%, 86.25% and 80.69%, respectively.

**Conclusion:** Fatigue correlated positively with stenosis progression. Factors associated with fatigue could contribute to better prediction for atherosclerosis progression.

## Introduction

Atherosclerotic plaque rupture is regarded as the clinical end-point event in the process of atherosclerosis progression. Stenosis is a common abnormal condition in arteries mainly due to atherosclerosis. Coronary gradual narrowing restricts blood flow, which causes ischemia and may induce heart attack. From a biomechanical perspective, vessel tissue fatigue is a chronic failure process induced by repetitive loading and could impact plaque development under the periodical arterial pressure (Bank et al., 2000; Ku & McCord, 1993; Stehbens, 1997; 2002). Plaque rupture can be considered as the result of accumulated fatigue damage (Versluis et al., 2006). Li et al. (2007) and his coworkers studied the fatigue crack with constructed two-dimensional model using *in vivo* magnetic resonance imaging (MRI) data (Pei et al., 2014). Huang et al. (2013) employed *in vivo* MRI-based 2D carotid model to study the development of crack and fatigue life. Their results showed that plaque without fibrous cap (FC) rupture or ulceration had a longer fatigue life compared with those with FC rupture or ulceration (*p* = 0.03).

Paritala et al. (2020) characterized the fatigue behavior of carotid arteries using uniaxial tensile test and provided an understanding of stress-relaxation and cyclic behavior. Bank et al. (2000) showed that fatigue is caused from cyclic stress by *ex-vivo* experiments, and found that fatigue is proportional to stress amplitude and mean stress. Gao et al. (2009) also pointed out that relative stress variation during a cycle in the fibrous cap is a potential indicator for plaque fatigue process by fluid-structure interaction (FSI) models of carotid arteries. The mainstream opinion is that stresses derived from periodical pressure is alternating stress, which is the main cause of fatigue.

Many researchers tried to find risk factors related to plaque progression. Using serial coronary computed tomography angiography, Won et al. used change of coronary plaque volume to measure plaque progression and explored the effects of the triglyceride glucose (TyG) index and body mass index (BMI) on plaque progression, respectively (Won et al., 2019; Won et al., 2020). Their studies have shown that BMI were not associated with plaque progression and TyG index had a positive and significant association with plaque progression (odd ratio = 1.409, confidence interval = [1.062–1.869], *p* = 0.017). Morphological factors, such as plaque composition and size, lumen size, fibrous cap thickness and others, may play significant roles in atherosclerosis progression. Ever since computational fluid dynamic (CFD) models have been used as a common tool to explore the mechanism of atherosclerosis progression and rupture, endothelial shear stress was found to be an important biomechanical factor for atherosclerosis progression (Vergallo et al., 2014). Corban et al. (2014) indicated that combining plaque burden, wall shear stress (WSS) and plaque phenotype was helpful to improve prediction accuracy of plaque progression. However, a study based on carotid atherosclerotic mouse model showed that WSS decreased strikingly during atherosclerotic progression, but the correlation between WSS and plaque area was weak and no statistical significance was found (*p* > 0.05) (Xing et al., 2018).

Besides, plaque fatigue would be a noteworthy factor in plaque progression. Stehbens (1997) hypothesized that atherosclerosis was the response to hemodynamically induced repetitive stresses due to the pulse pressure. Thondapu et al. (2017) pointed out that axial stress arises from longitudinal stretching of vessels exposed to cyclical blood flow and cardiac motion, and circumferential stress arises from hydrostatic pressure exerting outward radial force on vessels. The periodic pressure caused from pulsatile blood flow generates mechanical stresses. These mechanical factors contribute to plaque fatigue in an integrated manner and play a vital role in plaque progression (Wang L. et al, 2019; Guo et al., 2021).

In this paper, *in vivo* Virtual Histology intravascular ultrasound (VH-IVUS) data at baseline and follow-up were acquired from seven patients and used to construct thin-slice models for stress/strain calculations. Plaque fatigue was measured by stress/strain amplitudes in one cardiac cycle at baseline. The change of lumen area between baseline and follow-up was used as the measurement for atherosclerosis stenosis progression. Analyses for correlations between plaque fatigue and morphological characters and correlations predictors (morphological and biomechanical factors) and stenosis progression were performed. Machine learning approaches including random forest was employed to determine the prediction accuracy of plaque fatigue for predicting stenosis progression.

## Methods

### Virtual Histology-Intravascular Ultrasound Data Acquisition and Processing

Baseline and follow-up *in vivo* intravascular ultrasound (IVUS) and angiography data were acquired from seven participants (gender: 5M and 2F, average age: 59.2) at Cardiovascular Research Foundation (CRF) using protocol approved by the Institutional Review Board and informed consents were obtained from these patients. Patients were selected from a CRF data set where patients were with stable angina pectoris undergoing percutaneous coronary intervention (PCI). Patients with acute coronary syndrome, severe calcified lesion, chronic total occlusion or chronic kidney disease (Cr > 1.5 mg/dl) were excluded. Baseline data is the data set acquired at the first screening, which included IVUS, OCT, angiography, blood pressure, and general patient demographic information. These spans of the follow-up time for seven participants were 6–12 months (median 9 months). When electrocardiogram (ECG) signal was connected, VH-IVUS images were acquired automatically using Volcano S5 Imaging System (Volcano Crop., Rancho Cordova, CA, United States). Four tissue types were marked in color on VH-IVUS image: lipid-rich necrotic core in red, calcium in white, fibrous tissue in dark green and fibro-fatty tissue in light green. Segmentation of VH-IVUS images was performed by an in-house software package programmed in MATLAB (The MathWorks, Inc., Natick, MA, United States). The target vessel segment was selected based on angiography data. The registration of VH-IVUS images at baseline and follow-up from same vessel segment was executed using vessel branches which is the main landmark for location of vessel segment. These 305 paired VH-IVUS slices were matched and used for plaque modeling. Images generated at vessel bifurcations were excluded from this study.

### The Thin-Slice Model With Mooney-Rivlin Material Model

A 3D thin-slice modeling approach was adopted in this paper to obtain plaque stress/strain values. Thin slice models were selected since the model construction requires much less time (a few minutes per model) and is more suitable for potential clinical implementations (Wang L. et al, 2019). For each slice, the 3D thin-slice model was constructed by adding a thin slice thickness (0.5 mm, which is the spatial distance between two adjacent images generated during catheter pullback) to the 2D slice. For plaque models based on *in vivo* data, axial stretch is a non-negligible factor when calculating the stress/strain distribution in coronary (Thondapu et al., 2017). Axial shrinkage was set to be 5% in our models because atherosclerotic vessels were stiffer than healthy vessels. *In-vivo* VH-IVUS image was reconstructed with radiofrequency data captured at the peak of R-wave in ECG signal (Garcìa-Garcìa et al., 2011). The peak of the R-wave is commonly used to represent the end-diastole phase, so the acquired VH-IVUS data can be regarded as being generated at minimum arterial pressure. Hence circumferential shrinkage was applied in our models in order to make model shape under minimum pressure consistent with VH-IVUS. Pulsating arm pressure conditions were prescribed at lumen surface in thin-slice models. The construction of thin-slice models can be found in our previous publication (Guo et al., 2017). Lipid/calcification and other tissues were assumed to be isotropic and anisotropic, respectively. The strain energy density function of modified Mooney-Rivlin model for isotropic and anisotropic were Eqs 1, 2, respectively (Holzapfel et al., 2000):

$$W_{\mathrm{iso}}=c_{1}\left(I_{1}-3\right)+c_{2}\left(I_{2}-3\right)+D_{1}\left\{\exp\left[D_{2}\left(I_{1}-3\right)\right]-1\right\}$$ (1)

$$W_{aniso}=W_{\mathrm{iso}}+\frac{K_{1}}{K_{2}}\left\{\exp\left[K_{2}{\left(I_{4}-1\right)}^{2}-1\right]\right\}$$ (2)

Where $I_{1}=\sum C_{ij}$ , $I_{2}=\frac{1}{2}\left(I_{1}^{2}-C_{ij}C_{ij}\right)$ , $I_{1}$ and $I_{2}$ are the first and second invariants of right Cauchy-Green deformation tensor $C=\left[C_{ij}\right]=X^{T}X$ , $X=\left[X_{ij}\right]=\left[\partial x_{i}/\partial a_{j}\right]$ , $\left(x_{i}\right)$ is current position, $\left(a_{i}\right)$ is original position, $I_{4}=C_{ij}{\left(n_{c}\right)}_{i}{\left(n_{c}\right)}_{j}$ , $n_{c}$ is the unit vector in the circumferential direction of the vessel, $c_{1}$ , $c_{2}$ , $D_{1}$ , $D_{2}$ , $K_{1}$ and $K_{2}$ are material parameters.

The material parameters of lipid, calcification and other vessel tissues from existing literature were used (Guo et al., 2017): Lipid: $c_{1}$ = 0.5 kPa, $c_{2}$ = 0 kPa, $D_{1}$ = 0.5 kPa, $D_{2}$ = 1.5. Calcification: $c_{1}$ = 92 kPa, $c_{2}$ = 0 kPa, $D_{1}$ = 36 kPa and $D_{2}$ = 2. Other vessel tissues: $c_{1}$ = −278.7 kPa, $c_{2}$ = 24.35 kPa, $D_{1}$ = 133.7 kPa, $D_{2}$ = 2, $K_{1}$ = 7.19 kPa, $K_{2}$ = 23.5.

**FIGURE 1:**
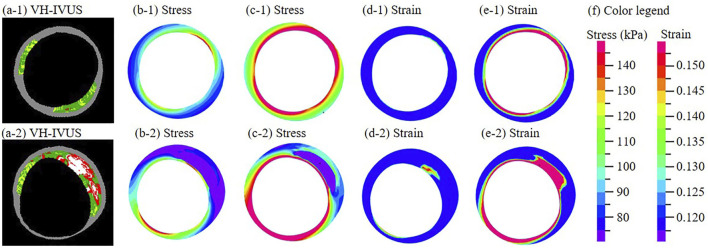
**(a-1)**–**(a-2)** One paired VH-IVUS images from the same location at baseline **(a-1)** and follow-up **(a-2)**. **(b-1)**−**(b-2)** Stress distribution of the thin-slice model at minimum pressure. **(c-1)**−**(c-2)** Stress distribution at maximum pressure. **(d-1)**−**(d-2)** Strain distribution at minimum pressure. **(e-1)**−**(e-2)** Strain distribution at maximum pressure. **(f)** Color legend. For sub-figures **(a-□)**−**(e-□)**, □ = 1 is for the baseline slice; □ = 2 is for the follow-up slice.

All models were solved by a finite element software ADINA (Adina R & D, Watertown, MA, United States) following our established procedures (Guo et al., 2017). Figure 1 shows distributions of stress and strain under maximum and minimum pressure conditions at baseline and follow-up. More details can be found from Guo et al. (2017).

### Measurements of Plaque Fatigue

The stress and strain of each slice were extracted from the solution of the thin-slice model. The stress and strain mentioned below refer to maximum principal stress and maximum principal strain. The values of stress and strain during cardiac cycle were extracted for each slice (2 × 305 = 610 slices in total).

Since plaque rupture usually occurs on luminal wall, the stress and strain at the location of superficial vascular wall was used in the following analysis. The stress amplitude and strain amplitude were defined as the stress variation and strain variation during one cardiac cycle, respectively. The stress and strain amplitudes were regarded as measurements for plaque fatigue in our study. The amplitude of average stress/strain on luminal wall and amplitude of maximum stress/strain on luminal wall during one cardiac cycle were all calculated to access plaque fatigue. These formulas are as follows:

$$\mathrm{Maximum}\;\mathrm{stress}\;\mathrm{amplitude}=\mathrm{maximum}\;\mathrm{stres}s_{\mathrm{at}\;\max\;\mathrm{pressure}}-\mathrm{maximum}\;\mathrm{stres}s_{\mathrm{at}\;\min\;\mathrm{pressure}}$$ (3)

$$\mathrm{Maximum}\;\mathrm{strain}\;\mathrm{amplitude}=\mathrm{maximum}\;\mathrm{strai}n_{\mathrm{at}\;\max\;\mathrm{pressure}}-\mathrm{maximum}\;\mathrm{strai}n_{\mathrm{at}\;\min\;\mathrm{pressure}}$$ (4)

$$\mathrm{Average}\;\mathrm{stress}\;\mathrm{amplitude}=\mathrm{average}\;\mathrm{stres}s_{\mathrm{at}\;\max\;\mathrm{pressure}}-\mathrm{average}\;\mathrm{stres}s_{\mathrm{at}\;\min\;\mathrm{pressure}}$$ (5)

$$\mathrm{Average}\;\mathrm{strain}\;\mathrm{amplitude}=\mathrm{average}\;\mathrm{strai}n_{\mathrm{at}\;\max\;\mathrm{pressure}}-\mathrm{average}\;\mathrm{strai}n_{\mathrm{at}\;\min\;\mathrm{pressure}}$$ (6)

### Measurements of Stenosis Progression

The cross-section area of lumen is an important index of vessels stenosis. The change of lumen area from baseline to follow-up was used as the measurement for stenosis progression (reduction in lumen area), which definition is as follows:

Delta lumen area(DLA)=Lumen area at baseline−lumen area at follow−up (7)

Positive DLA value means luminal narrowing from baseline to follow-up. All 305 slices at baseline were divided in two classes (label 0 and label 1) according to the non-positive or positive sign of DLA value. Label 0 class represents the set of slices with non-progressive luminal stenosis while label 1 class represents the set of slices with progressive luminal stenosis. Classification prediction of stenosis progression was performed by machine learning methods.

### Morphological and Biomechanical Factors Used as Predictors

Values of twelve biomechanical factors were extracted at baseline from thin-slice models. Those factors included maximum and average stress/strain amplitudes, maximum and average stress/strain at minimum and maximum pressure etc. and were prepared to be used as candidate predictors for stenosis progression. Seven morphological factors including plaque burden (PB) at minimum pressure, lumen/wall area at minimum and maximum pressure, and changes of lumen and wall from minimum pressure to maximum pressure [called lumen area amplitude (9) and wall area amplitude (10) for briefly] were also used as candidate factors.

PB=(plaque area/cross−sectional area of external elastic membrane)∗100% (8)

$$\mathrm{lumen}\;\mathrm{area}\;\mathrm{amplitude}=\mathrm{lumen}\;\mathrm{are}a_{\mathrm{at}\;\max\;\mathrm{pressure}}-\mathrm{lumen}\;\mathrm{are}a_{\mathrm{at}\;\min\;\mathrm{pressure}}$$ (9)

$$\mathrm{wall}\;\mathrm{area}\;\mathrm{amplitude}=\mathrm{wall}\;\mathrm{are}a_{\mathrm{at}\;\max\;\mathrm{pressure}}-\mathrm{wall}\;\mathrm{are}a_{\mathrm{at}\;\min\;\mathrm{pressure}}$$ (10)

### Correlation Analysis

Linear Mixed-Effects (LME) model was used to study the correlation between morphological factors, biomechanical factors and DLA. The correlation analysis of DLA and maximum stress amplitude was taken as an example to explain how the LME model was used in this study below.

The LME model was defined as

$$y_{ij}=\beta_{0}+\beta_{1}x_{ij}+b_{j}+\varepsilon_{ij}$$ (11)

where $y_{ij}$ is the DLA value on the i th slice of j th patient, $x_{ij}$ is the corresponding value of maximum stress amplitude. $\beta_{0}$ and $\beta_{1}$ are the fixed effects of DLA and maximum stress amplitude at baseline, respectively. $\varepsilon_{ij}$ is the random error terms which is assumed to follow a joint Gaussian distribution with mean 0.

The dependence-adjusted correlation coefficient r is given by

$$r={\hat{\beta }}_{1}\sqrt{\frac{\hat{var}\left(x\right)}{\hat{var}\left(y\right)}}$$ (12)

where ${\hat{\beta }}_{1}$ is the estimated slope coefficient by fitting maximum stress amplitude to DLA with the LME model, $\hat{var}\left(x\right)$ and $\hat{var}\left(y\right)$ are the sample variances.

The correlation analyses between plaque fatigue and morphological characters, predictors and stenosis progression were performed by R software (R 3.1.3, The R Foundation for Statistical Computing). The dependence-adjusted correlation coefficient was adopted to measure the dependence of variables and statistical significant was assumed if *p* < 0.05.

### Predictor Selection and Classification Prediction Using Random Forest

After 100 times testing using our data, our results indicated that the performance of random forest (RF) was better than that from the other machine learning methods (least square support vector machine (SVM), discriminant analysis and generalized linear mixed model). Therefore, RF was used as our prediction method in this study. The RF method uses multiple trees to train and predict the samples and uses the voting mechanism of multiple decision trees to resist the overfitting of decision trees. Training data consists of resampling n times with replacement from dataset (size equal to n). Some samples would not appear in training data because of sampling with replacement, which is called out-of-bag (OOB) data. OOB data is used as test set in our RF method.

Nineteen factors extracted from 305 slices (their values stored in a 305 × 19 matrix) were used as the input dataset for RF. The number of factors tried for splitting (Mtry) and the number of trees grown (Ntree) in the RF were two input parameters. Two parameters (Ntree and Mtry) of RF were optimized to guarantee high accuracy of classification prediction. The number of variables to be selected and tested for the best split when growing the trees (Mtry) was obtained by iteration based on OOB error. After Mtry value obtained, the number of decision trees to be generated (Ntree) would be set to minimum value that satisfies the error in RF model minimum and stable.

Dimensionality reduction of candidate factors were performed using “varSelRF” package. The mean decrease Gini index of factors were also calculated to ensure availability of factors selection. Gini index was defined as

$$\mathrm{Gini}\left(t\right)=1-\sum_{i=0}^{1}P{\left(i\right)}^{2}$$ (13)

where P(i) is the proportion of “lable i ” class in the dataset at the current node t. Gini impurity at node t was denoted as I(t) , then Mean Decrease Gini index was defined as

$$\mathrm{Mean}\;\mathrm{Decrease}\;\mathrm{Gini}\;\mathrm{index}=\sum_{Ntree}\sum_{t}\left(I\left(t\right)-\mathrm{Gini}\left(t\right)\right)$$ (14)

The classification prediction was implemented with “randomForest” package in R. The output from RF was a 305-dimensional binary vector. Cross validation or a separate accuracy assessment dataset is not necessary for RF algorithm, because the OOB error provides an unbiased estimate of error (Liaw & Wiener, 2002; Lawrence et al., 2006; Prinzie & Van den Poel, 2008). Therefore, the OOB error was adopted to estimate the misclassification error. Then confusion matrix was constructed to compare the true class with the class predicted by RF classifier and to calculate the overall accuracy. Sensitivity, specificity, positive and negative prediction values were also calculated as following.

Overall accuracy=(TP+TN)/(TP+FN+FP+TN) (15)

Sensitivity=TP/(TP+FN) (16)

Specificity=TN/(FP+TN) (17)

Positive prediction value=TP/(TP+FP) (18)

Negative prediction value=TN/(FN+TN) (19)

where TP is the number of true positive, FN is the number of false negative, FP is the number of false positive and TN is the number of true negative.

## Results

### Fatigue Correlated Positively With Lumen Area Amplitude and Negatively With Plaque Burden

The maximum and average stress/strain amplitude were regarded as the measurement of fatigue. In one cardiac cycle, there were a strong positive correlation between lumen area amplitude and fatigue and a negative correlation between PB and fatigue (Table 1). Especially, the correlation between average strain amplitude and lumen area amplitude was 0.3247 (*p* < 0.0001), and the correlation between average stress amplitude was −0.2808 (*p* < 0.0001).

**TABLE 1:** The correlation of morphological and biomechanical factors at baseline.

Morphological factors	Biomechanical factors	Correlation coefficient	*p* value
Lumen area amplitude	Maximum stress amplitude	0.0708	9.7 × 10^−6^
	Average stress amplitude	0.2103	2.3 × 10^−26^
	Maximum strain amplitude	0.1409	9.6 × 10^−61^
	Average strain amplitude	0.3247	1.3 × 10^−66^
PB	Maximum stress amplitude	−0.0992	3.6 × 10^−4^
	Average stress amplitude	−0.2808	2.0 × 10^−24^
	Maximum strain amplitude	−0.0888	0.007
	Average strain amplitude	−0.1786	9.7 × 10^−12^

### Fatigue Correlated Positively With Stenosis Progression

Factors that had a significant correlation with DLA were shown in Table 2. There were significant correlations between stenosis progression and maximum stress amplitude (*r* = 0.1313, *p* < 0.05), average stress amplitude (*r* = 0.3357, *p* < 0.05) and average strain amplitude (*r* = 0.5376, *p* < 0.05). In addition, the correlation between PB and DLA was best (*r* = −0.5729, *p* < 0.05).

**TABLE 2:** The correlation of baseline factors and stenosis progression.

Stenosis progression	Factors	Correlation coefficient	*p* value
DLA	Maximum stress amplitude	0.1313	0.0499
	Average stress amplitude	0.3357	0.0019
	Average strain amplitude	0.5376	0.0235
	Average stress at maximum pressure	0.3813	0.0199
	Average strain at maximum pressure	0.5613	0.0208
	PB	−0.5729	4.6 × 10^−6^

### RF Method Input Parameters

**FIGURE 2:**
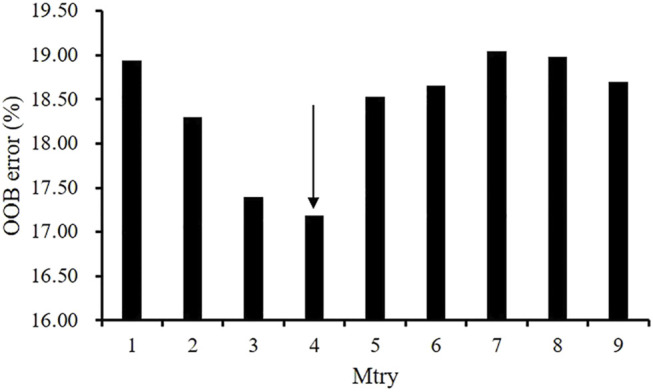
The effect of the number of variables tried at each split (Mtry) on the performance of RF using the OOB estimate of error (%).

**FIGURE 3:**
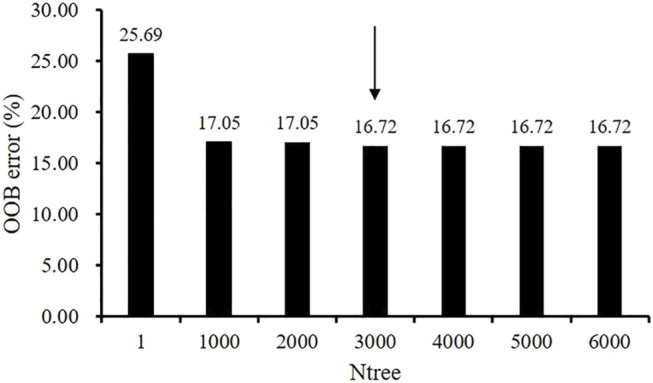
The effect of the number of trees (Ntree) parameter on the performance of random forest RF using the OOB estimate of error (%).

Before performing factors selection, it was essential to evaluate the effect of the two RF parameters (Mtry and Ntree) on the misclassification error. Figure 2 shows that Mtry = 4 was proved to be the best choice in terms of the OOB error rate (17.8%). When examining the Ntree parameter, results showed that OOB error rates were stabilized after 3,000 trees (Figure 3). Therefore, two parameters were set as Mtry = 4 and Ntree = 3,000 for all further analyses.

### Factors Selection

Predictor values obtained from all 305 slices were used as input variables into the RF algorithm using two determined parameters--Mtry and Ntree. Eight factors were selected by “varSelRF” package including average stress amplitude, wall area at minimum pressure and maximum pressure, lumen area at minimum pressure and maximum pressure, wall area amplitude, lumen area amplitude and PB. The mean decrease in Gini index as calculated by 19 factors was then used to rank the factors and check factors selection. Eight Factors selected from “varSelRF” package and ranking of mean decrease in Gini index are consistent. Tables 3, 4 shows mean decrease in Gini index of eight factors as calculated by RF. Results showed that the baseline PB had the highest mean decrease in Gini index. In addition, average stress amplitude was the only one mechanical factors in top eight factors.

**TABLE 3:** The value of mean decrease in Gini index for top eight factors.

Factors	Mean decrease in gini index
PB	15.03
Lumen area amplitude	13.38
Lumen area at maximum pressure	12.07
Lumen area at minimum pressure	11.88
Wall area at minimum pressure	11.67
Wall area at maximum pressure	11.43
Wall area amplitude	10.45
Average stress amplitude	8.81

**TABLE 4:** The confusion matrix showing the overall classification accuracy for two classes.

		Prediction	
		Non- narrowing	Narrowing
Ground truth	Non- narrowing	138	22
	Narrowing	28	117
Overall classification accuracy = 83.61%			

### Classification and Accuracy Assessment

The best predictor was the combination of PB, lumen area amplitude, lumen areas at maximum and minimum pressure, wall areas at maximum and minimum pressure, wall area amplitude and average stress amplitude. Using the combination of eight factors as the predictor and RF as the prediction method, the overall classification accuracy is 83.61%. The sensitivity (i.e., recall) is 86.25%, and specificity is 80.69%. Precision (i.e., positive prediction value) and negative prediction value is 83.13% and 84.17%, respectively. The overall OOB error rate for all the classes was 16.39% using the best predictor. For discriminating two classifications (progressive and non-progressive luminal stenosis), the confusion matrix shows that the label 0 class (non-progressive) has the lower error rate (13.75%), while the label 1 class (progressive) has the higher error rate (19.31%).

## Discussion

### Plaque Fatigue Factors Related to Plaque Progression

Even though there have been a number of publications using morphological, biomechanical and biochemical factors for atherosclerosis progression prediction in existing research, seeking key factors contributing to atherosclerosis progression is still a challenging problem (Saam et al., 2007; Wang et al., 2019; Won et al., 2019; Won et al., 2020). Wang et al. (2015) conducted IVUS-based fluid-structure interaction (FSI) modeling analysis to study the correlation between the biomechanical factors and morphological factors. Their results indicated that critical plaque wall stress (CPWS) correlated with minimum cap thickness and lipid percentage with *r* = −0.6414 and *r* = 0.2445 respectively (*p* < 0.0001). Although the correlation of CPWS and minimum cap thickness was strong, it remains to be confirmed by more studies based on high-resolution images since low resolution of IVUS image (∼150 μm) cannot capture the thin fibrous cap (<65 μm).

The studies mentioned above concentrated on plaque morphology and biomechanical factors extracted from one static moment, whereas amplitudes of morphology and biomechanics during one dynamic cardiac has gone unheeded. Vascular tissue fatigue occurs under cyclic stress and is related to atherosclerosis progression closely. The study of Bank et al. (2000) showed that fatigue is proportional to stress amplitude and mean stress by *ex-vivo* experiments. Our results indicated that average strain amplitude had a significant positive correlation with lumen area amplitude (*r* = 0.3247, *p* < 0.05) and average stress amplitude had a significant negative correlation with PB (*r* = −0.2808, *p* < 0.05). Our study also showed that fatigue has a positive correlation to stenosis progression according Table 2. Tables 2, 3 also show that the amplitudes of lumen area, wall area and average stress are significant factors for atherosclerosis progression, which improved our understanding for the relationship between plaque fatigue and stenosis progression.

### Plaque Progression Prediction Using Plaque Fatigue

Atherosclerosis progression could be measured by different plaque morphological parameters such as lumen area change, plaque burden change, plaque area change, plaque volume change, lipid percentage change, etc. (Guo et al., 2021; Saam et al., 2007; Wang Q. et al, 2019; Won et al., 2020; Won et al., 2019). Wang et al. used IVUS-based FSI models and found that the combination of plaque wall stress and wall shear stress is the optimal predictor for changes of plaque burden from baseline to follow-up with a prediction accuracy of 68.1% (Wang L. et al, 2019). Corban et al. (2014) found that combination of plaque burden, wall shear stress, and plaque phenotype has incremental value for prediction of coronary atherosclerotic plaque progression. Bourantas et al. (2020) defined atherosclerotic progression as a significant reduction in lumen (>7.5%) and increase in plaque burden (>8.8%) at follow-up, and gave the atherosclerotic progression prediction using multivariate linear regression models. Their study showed that area under the curve (AUC) using IVUS-based morphological predictors is 0.824 and the AUC is raised to 0.847 after adding WSS into predictor. A research group from Turkey employed different artificial neural network models to predict coronary stenosis, and their results were more than 71% for sensitivity, 76% for specificity and 81% for accuracy (Çolak et al., 2008). A large number of traditional statistical methods and machine learning methods have been used in atherosclerosis progression with different measurements, for instance, generalized linear mixed model, RF, SVM, neural network, etc. In fact, no matter which method we choose, we are faced with the problem of predictor selection, parameter tuning, and so on. In this study, we used RF algorithm with optimal performance on our dataset as the prediction method, employed change of lumen area as the measurement of stenosis progression, added plaque fatigue as a class of predictor, the evaluation of the effect of RF parameters is shown in Figures 2, 3. Overall classification accuracy was 83.61% in this study. Our previous study showed that AUC is 0.963 for the prediction of lipid percentage progression using multi-factors (plaque fatigue was not included) and SVM, but the specificity was 0.777 (sensitivity = 0.974) (Guo et al., 2021). In this study, the values of sensitivity (86.25%) and specificity (80.69%) are both above 80% and the difference between sensitivity and specificity is only 5.56%.

### Measurements of Plaque Fatigue From Medical Images and Models

Although plaque fatigue has been mentioned as one of the mechanism that might play an important role in plaque progression, the plaque fatigue information, such as ultimate tensile stress amplitude, critical mechanical condition and plaque fatigue life, have never been measured *in vivo* from existing technology with the real time information (Huang et al., 2013; Riou et al., 2014). Fatigue life is often divided into three periods: crack nucleation, crack propagation and final rupture, but the first two periods are silent in clinical symptoms and cannot be detected by medical image, and only rupture could be captured by medical imaging. Most researchers studied the plaque fatigue (especially dynamics of rupture) using crack propagation models (Versluis et al., 2006; Li et al., 2007; Huang et al., 2013; Pei et al., 2014). Our study used stress/strain amplitudes as the measurements of plaque fatigue in IVUS images without rupture. Compared with IVUS, intravascular optical coherence tomography (OCT) with high resolution (∼10 μm) is sufficient to obtain more accurate measurement of morphology of superficial coronary, but OCT has only 1–2 mm penetration depth not enough to detecting whole vessel wall (Jang et al., 2002). However, wall area calculated by the contour of outer vessel wall was an important morphological factor for prediction of stenosis progression in this study (Table 3). In the future, medical imaging technology with high resolution and strong penetration may detect morphological features accurately and then acquire accurate fatigue information from models that are important to predict stenosis progression.

### Limitations

Our study has the following major limitations: 1) Sample size. Only 305 slices from seven patients were used in our studies since it is challenging to obtain a large number of follow-up data with high quality IVUS and angiography images. Large-scale patient studies are needed to further validate our findings. 2) Each thin-slice model is essentially only one slice and could not include vessel curvature. This is a model limitation. 3) Modeling limitation. Thin-slice models used in this study only provided structure stress and strain. They could not provide hemodynamic information (such as flow shear stress) which is a limitation. Thin-slice models need much less man power to construct and could be more practical for potential clinical implementations. While stress/strain values from thin-slice models have modest errors (5–12% depending on the samples) compared to the full 3D FSI models, Wang et al. showed that correlation relationship from thin-slice models had an impressive 90.5% agreement rate compared to the results from 3D FSI models (Wang Q. et al, 2019). Clearly, it remains to be true that full 3D FSI models could be a better choice for more accurate stress/strain and wall shear stress calculations if model construction could be automated to reduce labor cost.

## Conclusion

Our preliminary results indicated that fatigue has a positive correlation with stenosis progression. Using eight morphological and biomechanical factors including fatigue, the overall classification accuracy, sensitivity and specificity of stenosis progression prediction with RF method were 83.61%, 86.25% and 80.69%, respectively. Factors associated with fatigue could contribute to better prediction for atherosclerosis progression.

## Data Availability

The original contributions presented in the study are included in the article/Supplementary Material, further inquiries can be directed to the corresponding authors.
